# Pharmacological Investigations in Traditional Utilization of *Alhagi maurorum* Medik. in Saharan Algeria: In Vitro Study of Anti-Inflammatory and Antihyperglycemic Activities of Water-Soluble Polysaccharides Extracted from the Seeds

**DOI:** 10.3390/plants10122658

**Published:** 2021-12-03

**Authors:** Fatma Zohra Chakou, Zakaria Boual, Mohamed Didi Ould El Hadj, Hakim Belkhalfa, Khaldoun Bachari, Zainab El Alaoui-Talibi, Cherkaoui El Modafar, Farah Hadjkacem, Imen Fendri, Slim Abdelkafi, Mounir Traïkia, Didier Le Cerf, Pascal Dubessay, Cédric Delattre, Guillaume Pierre, Philippe Michaud

**Affiliations:** 1 Laboratory for the Protection of Ecosystems in Arid and Semi-Arid Zones, Kasdi Merbah-University, Ouargla 30000, Algeria; fatmazohra.chakou@gmail.com (F.Z.C.); biozakaria1983@gmail.com (Z.B.); mohameddidi@yahoo.fr (M.D.O.E.H.); hadjkacemfarah@gmail.com (F.H.); 2 Institut Pascal, Université Clermont Auvergne, CNRS, Clermont Auvergne INP, 63000 Clermont-Ferrand, France; pascal.dubessay@uca.fr (P.D.); cedric.delattre@uca.fr (C.D.); guillaume.pierre@uca.fr (G.P.); 3 Scientific and Technical Research Center in Physicochemical Analysis, Tipaza 42000, Algeria; hakimbelkhalfa@gmail.com (H.B.); bachari2000@yahoo.fr (K.B.); 4 Faculty of Sciences and Techniques, University of Cadi Ayyad, Marrakech 40000, Morocco; zainab.elalaouitalibi@gmail.com (Z.E.A.-T.); elmodafar@uca.ac.ma (C.E.M.); 5 Laboratory of Enzymatic Engineering and Microbiology, Algae Biotechnology Team, National Engineering School of Sfax, Sfax University, Sfax 3038, Tunisia; slim.abdelkafi@enis.tn; 6 Laboratory of Plant Biotechnologies Applied to the Improvement of Plants, Faculty of Sciences, Sfax University, Sfax 3038, Tunisia; imen.fendri@fss.usf.tn; 7 Institut de Chimie de Clermont-Ferrand, Université Clermont Auvergne, CNRS, SIGMA Clermont, 63000 Clermont-Ferrand, France; mounir.traikia@uca.fr; 8 Département de Chimie, Université de Rouen Normandie, INSA Rouen, CNRS, PBS, 76000 Rouen, France; didier.lecerf@univ-rouen.fr

**Keywords:** *Alhagi*, Sahara, polysaccharides, galactomannan

## Abstract

The anti-inflammatory and antihyperglycemic effects of polysaccharides extracted from *Alhagi* *maurorum* Medik. seeds, spontaneous shrub collected in Southern of Algerian Sahara were investigated. Their water extraction followed by alcoholic precipitation was conducted to obtain two water-soluble polysaccharides extracts (WSPAM1 and WSPAM2). They were characterized using Fourier transform infrared, ^1^H/^13^C Nuclear Magnetic Resonance, Gas Chromatography-Mass Spectrometry and Size Exclusion Chromatography coupled with Multi-Angle Light Scattering. The capacity of those fractions to inhibit α-amylase activity and thermally induced Bovine Serum Albumin denaturation were also investigated. WSPAM1 and WSPAM2 were galactomannans with a mannose/galactose ratio of 2.2 and 2.4, respectively. The SEC-MALLS analysis revealed that WSPAM1 had a molecular weight of 1.4 × 10^6^ Da. The investigations highlighted antinflammatory and antihyperglycemic effects in a dose-dependant manner of WSPAM1 and WSPAM2.

## 1. Introduction

Natural polysaccharides possess chemical structures, making them a key source of lead compounds for management of critical diseases such as diabetes and inflammations [1]. They are also effective ways to prevent low side effects of currently available synthetic drugs e.g., acarbose, miglitol, and voglibose for treatment of diabetes and have noticeable healthy effects on hypoglycemia and gastrointestinal troubles [2,3,4]. They have demonstrated anti-inflammatory effects such as steroid and non-steroid drugs used to treat osteoporosis, hypertension, and cushing syndromes of the former and immunosuppression [5]. Thus, polysaccharides could be the next future alternate anti-diabetic [6,7,8] and anti-inflammatory [1,9] agents. The mechanisms involved in antidiabetic activity are numerous. They comprise the protection of pancreatic islets from harm by scavenging the free radicals and repairing destroyed β-cells [10]. They can also improve insulin secretion capacity of pancreatic β-cells [11]. The viscosity of polysaccharides can inhibit glucose diffusion [12,13] maintaining low levels of glucose concentration in the small intestine [12,14]. They are inhibitors of α-d-glucosidases in the bowel, resulting in reduction of carbohydrates’ decomposition and absorption [15,16,17]. Plant-derived polysaccharides have shown their anti-inflammatory activities through the modulation of inflammatory mediators and several immune cells [18]. They ameliorate the dysregulation of pro/anti-inflammatory cytokines [19]. They suppress the release of nitric oxide, Tumor Necrosis Factor-alpha and the expression of inducible nitric oxide synthase [20].

*Alhagi maurorum* (Fabaceae) is a spiny up shrub [21], native to South East Europe, North Africa, the Middle East and [22]. It has numerous appellations such as Shprim, Shook, Aqool, Lehlah and Shooq El Jamal [23], and therapeutic properties e.g., to remove kidney stones, ureter relaxer, as laxative, diaphoretic, diuretic and expectorant agent [24]. Regarding the literature, numerous phytochemical constituents have been identified from the plant such as glycosides, flavonoïds, alkaloïds, saponins, tannins and steroïds, followed by multiple pharmacological activities as antibacterial, anti-inflammatory, antipyretic, analgesic, anti-oxidant and diuretic ones [25].

To our knowledge, no study deals with the pharmacological potentials and chemical elucidation of water-soluble polysaccharides from *Alhagi maurorum* Medik. seeds.

## 2. Materials and Methods

### 2.1. Plant Material

*Alhagi maurorum* Medik. (Conservatoire et Jardin botaniques de la Ville de Genève and South African National Biodiversity Institute, Pretoria; Tela Botanica number: 61615), was harvested in August 2019 in Belbachir city for GPS coordinates 26°28′ north latitude and 8°28′ east longitudes of Illizi area (Southern of Algerian Sahara). The plant seeds were obtained manually from cloves previously dried at room temperature for two weeks. They were conserved in Kraft paper and preserved for further research.

### 2.2. Extraction and Purification of Polysaccharides

Two polysaccharide fractions were obtained from the water extraction of the plant seeds to confirm the monosaccharide composition of seed coats mucilage and endosperm cell walls. Firstly, 60.17 g of crushed material seeds were depigmented and defatted with acetone 96% under stirring for 14 h at 27 °C, then filtrated and replaced by ethanol 96% under reflux for 2 h and agitation at 80 °C. After filtration through a sintered glass filter of porosity 1 (100–160 μm), pellets were macerated three times in ultrapure water (1/10; *w*/*w*) under reflux and agitation (80 °C, 2 h). The mixture was centrifuged (20,000× *g*; 20 min; 20 °C) and the supernatant filtered under vacuum through a sintered glass filter of porosity 1 (100–160 μm). The filtrate was concentrated to a small volume under reducing pressure using a rotary evaporator followed by alcohol precipitation by three volumes of cold isopropanol 96% for overnight (−20 °C). After centrifugation (12,000× *g*, 20 min, 20 °C), the pellets were dissolved again in ultrapure water and supplemented by three volumes of isopropanol (96%) for 24 h at −20 °C. Thus, the precipitate containing polysaccharides extract was recovered by centrifugation (12,000× *g*, 20 min, 20 °C), coded WSPAM1 and dried using a freeze drier.

Secondly, 20 g of non crushed seeds were extracted in ultrapure water during 2 h at 40 °C under stirring, filter, dried at 45 °C, powdered and macerated two times in ultrapure water (1/10; *w*/*w*) under reflux and agitation (80 °C, 2 h). The same loop of twice alcoholic precipitation, dissolution was performed as described above to obtain the fraction WSPAM2.

### 2.3. Biochemical Composition 

The total and neutral sugar contents of WSPAM1 and WSPAM2 were quantified according to phenol-sulfuric acid [26] and resorcinol-sulfuric acid [27] assays, respectively, using glucose as standard [26,27]. Galacturonic acid was used as standard for m-hydroxydiphenyl assay [28] to determine uronic acid content. Phenolic compounds were quantified by the Folin–Ciocalteu method using gallic acid as standard as described by Singleton [29]. Bradford [30] assay was used to measure proteins content with Bovin Serum Albumin (BSA) as standard. Each analysis was done in triplicate, and data are expressed as means (±SEM).

### 2.4. FT-IR Spectroscopy Analysis

Characteristic prints of polysaccharides extracts (fifty scans) were recorded at room temperature (reference against air) on a VERTEX 70 FT-IR apparatus equipped with an ATR A225 diamante (Bruker VERTEX 70, Ettlingen, Germany) ranging from 4000 to 400 cm^−1^. The data were treated using OPUS 7.2 software (Bruker, Ettlingen, Germany).

### 2.5. Analysis of Monosaccharide Composition Using GC/MS-EI

Ten mg of each polysaccharide were hydrolyzed with 1 mL of TFA 2 M at 120 °C during 90 min, then evaporated under dryness using nitrogen stream at 60 °C. Derivatization of monosaccharides was realized according to Pierre et al. [31,32], dissolving the lyophilizate in pyridine and Sylon (BSTFA/TMCS; 99%/1%). The mix was incubated at room temperature 2 h and under Argon to form trimethylsilyl-O-glycosides which were solubilized in dichloromethane after evaporation of reagent. The samples were injected on an Agilent 6890 Series GC System coupled to an Agilent 5973 Network, equipped with an OPTIMA-1MS column (Macherey-Nagel; 30 m, 0.32 mm, 0.25 µm) from Macherey–Nagel with a helium flow rate of 2.3 mL/min. The first time, temperature was at 100 °C for 3 min. In a second step an increment of 8 °C/min up to 200 °C for 1 min was applied before a final increment of 5 °C/min up to 250 °C. Electronic Impact (EI, 70 eV) ionization method was performed with the trap temperature set at 150 °C and the target ion was fixed at 40–800 *m*/*z*. The relative molar proportions were calculated using area normalization method. As monosaccharides standards, Ara, Rha, Gal, Glc, Xyl, Man, GlcA and GalA were prepared and analyzed following the same procedure.

### 2.6. Polysaccharide Hydrolysis

One g of WSPAM1 was hydrolyzed in 100 mL of 20% H_2_SO_4_ at 50 °C during 30 min with stirring. The hydrolysate was neutralized with 10 M NaOH and then dialyzed (3.5 kDa) against ultrapure water for 72 h before being freeze-dried and coded HPAM.

### 2.7. NMR Analysis

WSPAM1 was dissolved at 100 g/L in D_2_O (99.9% D) and freeze-dried (three times). Before analysis, WSPAM was dissolved in D_2_O (100 g/L). The ^1^H NMR spectra were obtained at 353 K on a Bruker AVANCE III HD 500 MHz spectrometer equipped with Bruker 5 mm inverse probe TXI (^1^H/^13^C/^15^N) with z-gradient coil probe. For all samples, a one-dimensional spectrum (^1^H NMR) was acquired using a ZG sequence. 128 scans were collected with an 90° impulsion time of 9.7 µs at a power of 14 W, a 4 s relaxation time, an acquisition of 3.3 s, a spectral window of 20 ppm and 65 K data points zero-filled to 131 K before Fourier transformation with 0.3 Hz line broadening. The ^13^C NMR spectra were registered at 353 K on the same spectrometer as ^1^H spectra. Dimensional ^13^C NMR spectra were acquired using a ZGPG sequence (WALTZ16 power gated decoupling with a 80 µs at 0.2 W). 20,000 scans were collected with an 90° impulsion time of 12 µs at a power of 190 W, a 2 s relaxation time, an acquisition of 1.20 s, a spectral window of 220 ppm and 65 K data points zero-filled to 131 K before Fourier transformation with 3 Hz line broadening. 

### 2.8. Molecular Weight

High pressure size exclusion chromatography (HPSEC) equipped with on line three detectors, a He–Ne laser at 690 nm (HELEOS II, Wyatt Technology Corp, Santa Barbara, CA, USA), a multi-angle laser light scattering (MALLS) filled with a K5 cell (50 L), a differential refractive index (RID 10A, Shimadzu, Japan) and a viscosimeter (Viscostar II, Wyatt Technology Corp., USA) was carried out to study the molecular weight and homogeneity of polysaccharide extract, measuring parameters such as the weight average molecular weight (M_w_), the number-average molecular weight (M_n_) and the polydispersity index (Đ = M_w_/M_n_) of WSPAM1. Two columns (OHPAK SB-G guard column, OHPAK SB806 and 804 HQ columns (Shodex)) were eluted with LiNO_3_ 0.1 M at 0.5 mL/min. WSPAM1 was solubilized at 1 g/L in LiNO_3_ 0.1 M during 48 h at room temperature under stirring, and the solution was filtered through a 0.45 μm filter before injection through a 500 μL full loop.

### 2.9. Biological Properties of Polysaccharides Extracts 

#### 2.9.1. Albumin Denaturation Inhibitory Activity 

The anti-inflammatory test was carried out following Osman et al. [33] and Bakka et al. [34] with some modifications, to test their ability to inhibit the thermally denaturation of BSA. The polysaccharides extracts and Ibuprofen (control positive) were prepared at concentrations of 0.1, 0.2, 0.4, 0.8 and 1% (*w*/*v*). The react mixture contained 0.45 mL of BSA 0.5% (*w*/*v*), 0.05 mL of polysaccharide or Ibuprofen concentrations, excepted the negative control. The sample was incubated at 37 °C for 20 min and then heated at 70 °C for 10 min. After cooling, 2.5 mL of Phosphate Buffer Solution (pH 7.4) was added to the reaction mixture before measuring A_660_. The inhibition of albumin denaturation was calculated following Equation (1). The tests were carried out in triplicate.

(1) $$\mathrm{Inhibition}\;\left(\%\right)\;=\;\left(1-\frac{D}{C}\right)\;\times 100$$

*D* A_660_ of the test sample

*C* A_660_ of negative control (reading without inhibitor)

#### 2.9.2. Antihyperglycemic Activity

The control of glucose production following the consumption of food sources, such as α-amylase inhibitors, might be a good approach for the management of type 2 diabetes as well as hyperglycemia [35,36], in which WSPAM1, WSPAM2 and HPAM were investigated by evaluating inhibition of α-amylase activity as described by Kumar et al. [37] and Kajaria et al. [38], using their methods with minor modifications. Sixty μL of different aqueous solutions (1.25, 2.5, 3.75, 5, 6.25, 7.5, 8.75 and 10 mg/mL) of polysaccharide and acarbose (positive control) or ultrapure water (negative control) were mixed with 30 μL of α-amylase from human suffered of pancreatitis (5 IU/L) and incubated at 37 °C during 15 min. Subsequently, 150 μL (0.5 mg/mL) of 2-chloro-*p*-nitrophenyl-α-d-maltotrioside (CNPG3) used as substrate was added under stirring. Inhibition releasing of 2-chloro-*p*-nitrophenol measured polysaccharide’s ability to inhibit α-amylase activity measuring A_405_ and expressed according to Equation (2). The tests were carried out in triplicate.

Inhibition (%) = (Control_test_ − Test_sample_/Control_test_) × 100 (2)

### 2.10. Statistical Analysis

The results data were treated via Statistical Package for Social Science (SPSS) version 16 and Microsoft Excel 2007 for scheming graphs. The correlation between parameters was determined by Student’s *t*-test for conditions to use are *p*-value less than chosen significance level α = 0.05.

## 3. Results and Discussion

### 3.1. Biochemical Characterization

The isolated polysaccharides from *A. maurorum* Medik. seeds were amorphous white powders with a cream tint. The extraction yields of WSPAM1 and WSPAM2 were, respectively, 12.58% and 9.83% (*w*/*w*), which was higher than yields polysaccharides found from other seeds species of *Alhagi* genus, *Alhagi pseudalhagi* (MB) Desv. at 2.8% [39] and 4% of *Alhagi persarum* Boiss [40]. The biochemical analysis (Table 1) of WSPAM1 and WSPAM2 showed a similar biochemical composition. The polysaccharides extracts WSPAM1 and WSPAM2 were mainly composed of neutral sugars with values of 58.98 and 53.1% (*w*/*w*) respectively and low uronic acid contents with values of 12.76 and 13.75% (*w*/*w*), respectively. As well, both fractions are lightly contaminated by phenolic compounds (7.57% and 9.21% *w*/*w*) and very low protein contents (2.32% and 1.6% *w*/*w*) were measured. WSPAM1 was then chosen for structural investigations.

### 3.2. Structural Characterization

#### 3.2.1. FT-IR Spectroscopy

**Figure 1:**
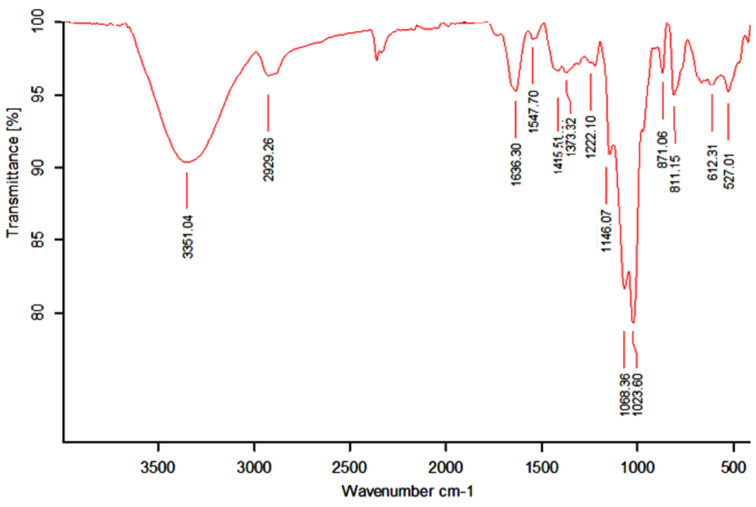
FT-IR spectrum of WSPAM1.

The infrared spectrum of WSPAM1 is represented in Figure 1. Characteristic bands of polysaccharides were attributed between 527 and 3351 cm^−1^. The intense peak at 3351.04 cm^−1^ corresponded to O-H stretching vibration from polysaccharide and water whereas the signal attributed at 2929.26 cm^−1^ was assigned to aliphatic bending groups (C-H) [41]. The vibration of carboxylate groups (C=O) were also observed at 1636.30 cm^−1^ [42]. The signals from 1222.10 to 1547.70 cm^−1^ were assigned to C-H deformation vibrations and rotational vibrations of C-OH groups [43].

The main peak at 1146.07 cm^−^^1^ was attributed to C-O functions of carbohydrates [44] while the two bands at 1023.60 and 1068.36 cm^−^^1^ suggested a pyranose form for carbohydrates constituting WSPAM1 [45]. The absorption bands at 871.06 cm^−1^ and 811.15 cm^−1^ were attributed, respectively, to α-glycosides linkages [46] and α-d-galactopyranose residue [47].

#### 3.2.2. GC-MS/EI 

**Figure 2:**
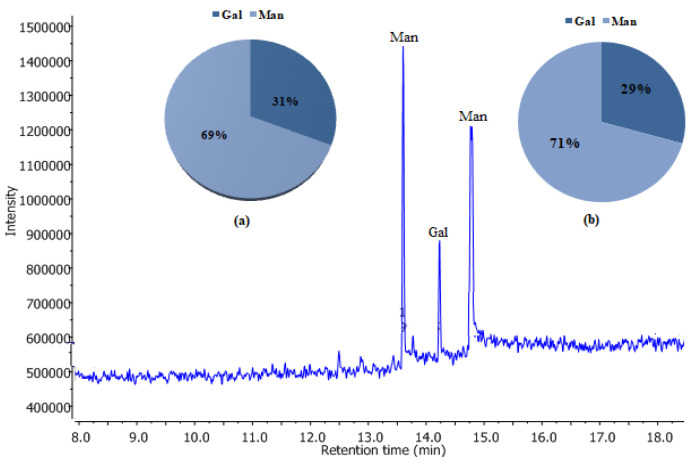
Monosaccharide compositions of WSPAM1 (**a**) and WSPAM2 (**b**) using GC-MS/EI.

As illustrated in Figure 2, GC-MS/EI analysis indicates that polysaccharide extracts WSPAM1 (a) and WSPAM2 (b) were exclusively composed of galactose and mannose with a ratio of Mannose/Galactose equal to 2.22 and 2.44, respectively. The pyranose form of monosaccharides was confirmed according to Harris et al. [48] with the estimation of the abundance of 204/217 ratio (*m*/*z*). These results suggested a galactomannan type polysaccharide and confirm the similarities of WSPAM1 and WSPAM2 compositions. These ratios were close to that reported for polysaccharides extracted from the seeds of Leguminosae [49], *A. persarum* Boiss (2.00) [40] and *Sonoran mezquite* (2.05) [50]. Kodiralieva and Rakhmanberdyeva [39] reported a lower M/G ratio of 0.5 for the polysaccharide extracted from *A. pseudalhagi* (MB) Desv seeds. However, the galactomannan nature of this polysaccharide should be questioned as authors identified significant amounts of other monosaccharides in its composition such as arabinose, xylose and rhamnose.

#### 3.2.3. ^1^H, ^13^C NMR and HSQC Analysis

**Figure 3:**
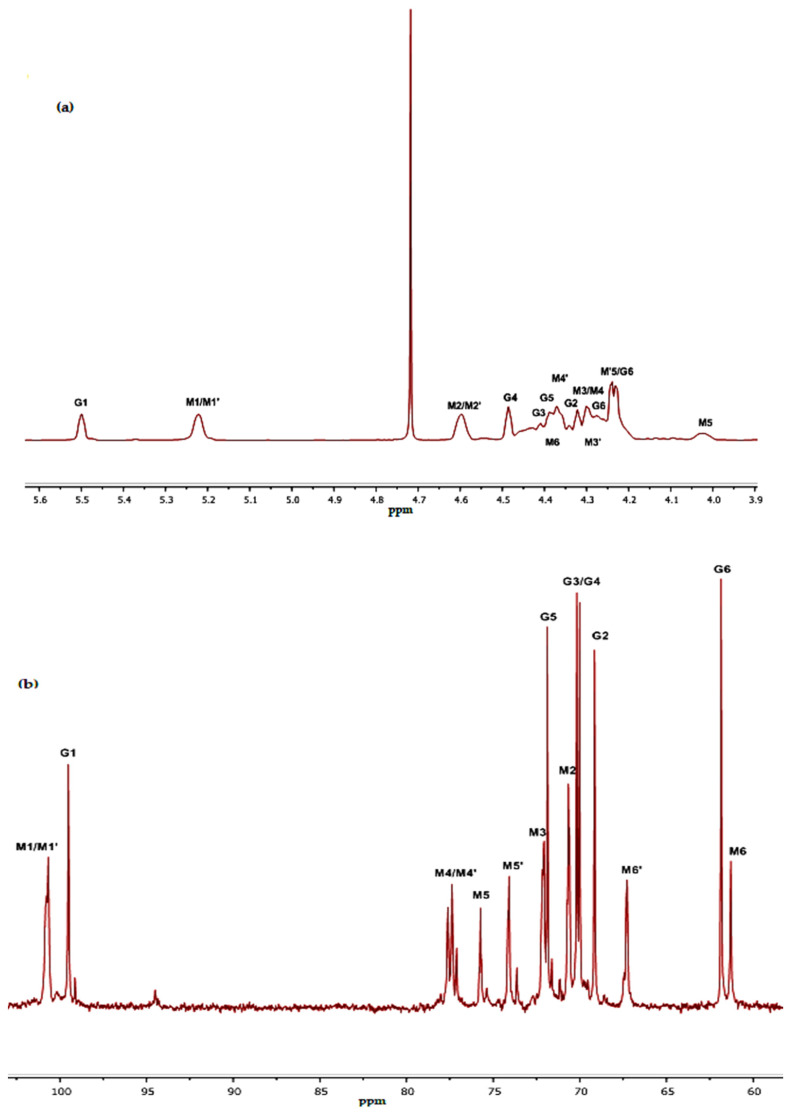
Spectra ^1^H NMR (**a**) and ^13^C NMR (**b**) of WSPAM1 from *Alhagi maurorum* Medik. seeds. G: α-d-galactopyranose; M: β-d-mannopyranose non substituted; M’: β-d-mannopyranose substituted at O-6 position.

The polysaccharide obtained from *Alhagi maurorum* (WSPAM1) was analyzed by ^13^C and ^1^H NMR spectroscopy. Analysis of the polysaccharide revealed specific carbohydrates signals of galactomannans confirming the monosaccharidic composition (Figure 3 and Table 2).

^1^H NMR spectrum showed two distinct signals at 5.51 and 5.23 ppm attributed to anomeric protons H-1 of α-d-galactopyranose (G1) and H-1 of β-d-mannopyranose (M1/M1’), respectively. The molar ratio of M/G calculated from the integrations of the relative peak areas of these two signals was 1.96. This value was coherent with that obtained after GC-MS analysis (M/G = 2.22).

As shown in ^13^C NMR spectrum (Figure 3b), the anomeric signal at 99.51 ppm and 100.67 ppm was assigned to C-1 of α-d-galactopyranose and unsubstituted β-d-mannopyranose residues, respectively. Moreover, signals observed at 61.85 ppm, 61.29 ppm and 67.27 ppm were attributed to C-6 of α-d-galactopyranose (G6), C-6 of an unsubstituted β-d-mannopyranose (M6) and C-6 of a substituted β-d-mannopyranose (M’6) in the same order.

Compared to other galactomannans extracted from the seeds of different leguminous plants, M/G ratio of WSPAM1 was slightly elevate to those estimated for *Astragalus gombo* Bunge, *Sesbania virgata* and *Leucaena leucocephala* to 1.7, 1.5 and 1.6, respectively [50,51,52,53]. The purity of galactomannan and the M/G ratios of seeds from *Fabaceae* was related to plant origin and extraction processes [40,53,54].

#### 3.2.4. Molecular Weight 

The molecular weight (M_w_ g/mol), molecular weight distribution (M_n_ g/mol) and intrinsic viscosity [η] of WSPAM1 extracted from the seeds of *A. maurorum* Medik. were determined using Size Exclusion Chromatography coupled with Multi-Angle Light Scattering (SEC-MALLS) (Table 3). The M_w_ and M_n_ were 1.40 × 10^6^ and 2.7 × 10^5^ g/mol, respectively. These values were elevated but coherent compared to other galactomannans reported in literature. As an example, the galactomannan obtained from *Astragalus gombo* Bunge seeds had a Mw of 1.1 × 10^6^ and a Mn of 0.82 × 10^5^ g/mol [51]. Moreover, Mw of galactomannans from *Delonix regia* and *Caesalpinia gilliesii* were, respectively, 7.23 × 10^5^ and 1.17 × 10^5^ g/mol [55,56]. It is well recognized that the molecular weight of galactomannans can be highly diversified depending on parameters such as their geographical and origins botanical, the fractionation and extraction conditions (e.g., pH, temperature, type of organic solvents used for aggregation) and the extent of the galactosyl substitution or depolymerization [57].

The hydrodynamic radius (R_h_) of 57.3 nm and the elevated polydispersity index of Đ = 5.2 indicated the high degree of polydispersity of WSPAM1, higher to those reported for several leguminous species in the range of 1.23 to 2.79 [55,56,58,59]. The intrinsic viscosity [η] was estimated at 970 mL/g which is in accordance with that found for *Astragalus gombo* Bunge galactomannan (860 mL/g) [51].

### 3.3. Biological Activities

#### 3.3.1. Inhibition of BSA Denaturation 

**Figure 4:**
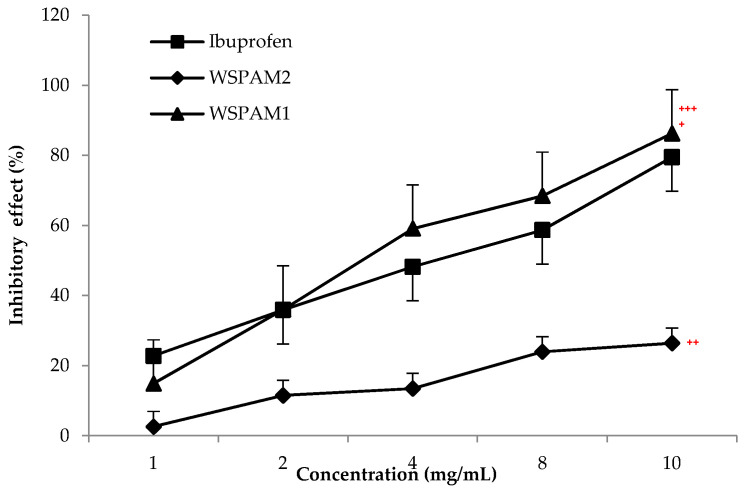
Inhibitory effects of WSPAM1, WSPAM2 and Ibuprofen on BSA thermally denaturation. ^+^ *p* > 0.1 and ^++^ *p* < 0.01 compared to Ibuprofen. Correlation between the inhibitory effect of polysaccharide extracts and the molecular weight was expressed with ^+++^ *p* < 0.001.

Inflammation is the body’s first immune system response to an infection, stress and damage. Continuous inflammation has a close relationship to many diseases, such as arthritis, atherosclerosis and cancer [18]. Regarding the literature, protein denaturation caused by some lysosomal enzymes released during the inflammation [33,60], is one of the biggest causes of biological loss of protein function [61]. The capacity of WSPAM1 and WSPAM2 on inhibition of BSA against heat denaturation temperature at 70 °C is shown in Figure 4. WSPAM1 has shown a higher inhibitory effect (14.89–86.23%) than the standard drug (Ibuprofen) (22.78–79.46%) at same concentrations from 1 to 10 mg/mL and IC_50_ values of 5.8 mg/mL and 6.29 mg/mL, respectively. However, a lower inhibitory activity was observed for WSPAM2 (2.59–26.42%), which was 3 times lower than other fractions at the same concentration range. This lower activity highlights the importance of the depigmented and defatted steps before the extraction of galactomannan from the seeds of *A. maurorum* Medik. Indeed, Ibanoglu (2005) reported that the augmentation in the enthalpy of denaturation of BSA in the presence of hydrocolloids was recognized to protect globular proteins against aggregation through blockage of their hydrophobic binding sites by the bulky polysaccharide moiety [62]. In addition, it is known that the biological activities of polysaccharides are markedly influenced by their molecular weights, monosaccharide compositions, glycosidic bond types and non sugar contents [63].

#### 3.3.2. Antihyperglycemic Activity

**Figure 5:**
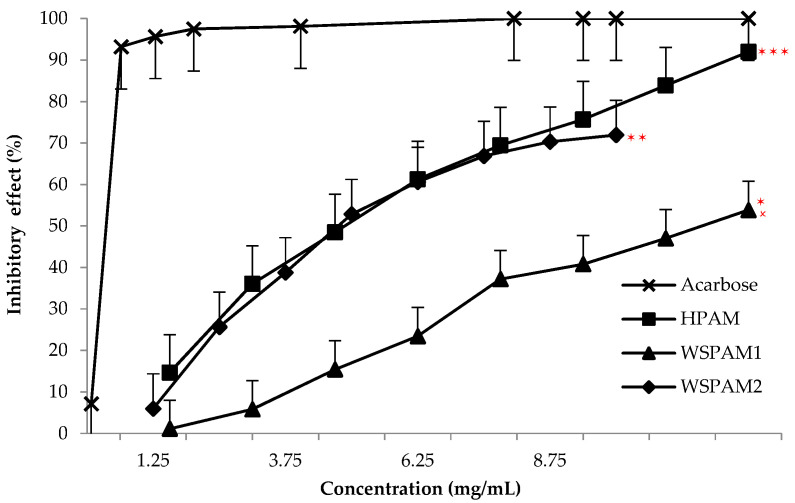
Antihyperglycemic activity of polysaccharide extracts of *A. maurorum* Medik. seeds on inhibition of α-amylase enzyme activity. ^✶^ *p* < 0.001, ^✶✶^ *p* < 0.05, ^✶✶✶^ *p* < 0.1 compared with acarbose (positive control) as well as to to molecular weight (^🗴^ *p* < 0.0001).

In vitro antihyperglycemic effects of polysaccharide extracts of *A. maurorum* Medik. seeds were quantified and compared to acarbose as positive control measuring inhibition of α-amylase activity (Figure 5). 

The results showed that the hydrolyzate HPAM (*p* < 0.1) and WSPAM2 (*p* < 0.05) significantly (*p* < 0.0001) have a strong inhibitory effect on α-amylase activity to 91.96 and 71.92% at 10 mg/mL with an IC_50_ values of 5.43 mg/mL and 6.81 mg/mL, respectively (Table 4). While a moderate significant inhibitory potential was attributed to WSPAM1 (*p* < 0.001) with a value of 53.83% (IC_50_ = 9.28 mg/mL) compared to 100% from acarbose to 6.45 mg/mL used of the drug. 

The inhibitory effects of polysaccharides extracted from the plant seeds were considerably better than other plants tested for their hypoglycemic activity and highly significantly correlated to the molecular weight (*p* < 0.0001). Indeed, according to Kabat and Bezer [64] most polysaccharides with medicinal properties were high molecules with molecular weights above 100 kDa. In addition, authors [16] reported a good inhibitory effect of α-glucosidase by a polysaccharide (APS) extract from dried *Radix Astragali* having a high molecular weight (693 kDa). Other polysaccharides obtained from *Cucurbita moschata* inhibited 41.3% of α-amylase activity at a concentration of 5 mg/mL [35]. A galactomannan extracted from the endosperm of *Adenanthera pavonina’s* L. seeds (GAP) exhibited after 30 days of treatment, at 1% and 2%, a significant glycemia decrease in comparison to diabetic control on streptozotocin induced diabetic mice [65]. In addition, galactomannan obtained from *Arenga Pinnata* Merr. significantly reduced blood glucose after 90 min treatment without any side effect [66]. Studies report that viscosity and concentration of polysaccharides contribute directly to their hypoglycemic effects because they limit digestive enzymatic activity and glucose absorption in the gut [67,68].

## 4. Conclusions

The structural characterization and in vitro biological investigations of water-soluble polysaccharides extracted from *A. maurorum* Medik. seeds revealed a galactomannan of high molecular weight close to 1.40 × 10^6^ g/mol with polydispersity value of 5.2. WSPAM1 had a significant inhibitory effect on BSA thermally denaturation with an IC_50_ = 5.8 mg/mL. Moreover, HPAM (hydrolyzed fraction) exhibited the strongest inhibitory α-amylase activity of 91.96% (IC_50_ = 5.3 mg/mL). Both in vitro studies of biological activities of WSPAM1 were correlated significantly to their molecular weights. Further in vivo and in vitro studies will be carried out to understand the specific inhibitory mechanisms of antihyperglycemic effects of polysaccharides extracted from the seeds of *Alhagi maurorum* Medik.

## Figures and Tables

**Table 1:** Biochemical characterization of polysaccharides from *A. maurorum* Medik. seeds.

	Carbohydrate Composition (*w*/*w* %)			Phenolic Compounds (*w*/*w* %)	Proteins (*w*/*w* %)
	Total	Neutral	Uronic Acids		
WSPAM1	49.21 ± 0.037	58.98 ± 0.02	12.76 ± 0.029	7.57 ± 2 × 10^−5^	2.32 ± 10^−3^
WSPAM2	43.95 ± 0.026	53.1 ± 0.009	13.75 ± 0.005	9.21 ± 0.5 × 10^−5^	1.6 ± 5 × 10^−4^

**Table 2:** Assignments of ^1^H/^13^C NMR signals of *Alhagi maurorum* seeds galactomannan.

	Chemical Shifts (ppm)					
Type of Unit	H/C-1	H/C-2	H/C-3	H/C-4	H/C-5	H/C-6
α-d-galactopyranosyl (G)	5.51/99.51	4.32/69.15	4.41/70.17	4.50/70.01	4.39/71.87	4.25/61.85
β-d-mannopyranosyl unsubstituted (M)	5.23/100.67	4.61/70.66	4.30/72.16	4.31/77.34	4.03/75.73	4.38/61.29
β-d-mannopyranosyl substituted (M’)	5.23/100.77	4.61/70.66	4.30/72.16	4.36/77.50	4.22/74.08	4.31/67.27

**Table 3:** Physicochemical analysis of WSPAM.

M_w_ (g/mol)	M_n_ (g/mol)	Polydispersity Index (Đ)	Intrinsic Viscosity [η] (mL/g)	R_h_ (nm)
1.40 × 10^6^	2.7 × 10^5^	5.2	970	57.3

**Table 4:** The median concentrations (IC50) of *A. maurorum* polysaccharides on inhibition of α-amylase activity.

	WSPAM1	WSPAM2	HPAM	Acarbose
IC50 (mg/mL)	9.28	6.81	5.43	6.45

## Data Availability

Raw data are available on request from the corresponding author.
